# Estropause, Sex Hormones and Metal Homeostasis in the Mouse Brain

**DOI:** 10.3389/fneur.2022.841822

**Published:** 2022-05-11

**Authors:** Tianbing Liu, Richard L. Bowen, Andrea C. Wilson, Craig S. Atwood

**Affiliations:** ^1^Institute of Pathology, Case Western Reserve University School of Medicine, Cleveland, OH, United States; ^2^Department of Pathology and Laboratory Medicine, University of Wisconsin Madison School of Medicine and Public Health, Madison, WI, United States; ^3^OTB Research, Charleston, SC, United States; ^4^Division of Geriatrics and Gerontology, Department of Medicine, University of Wisconsin Madison School of Medicine and Public Health, Madison, WI, United States; ^5^Geriatric Research, Education and Clinical Center, Veterans Administration Hospital, Madison, WI, United States; ^6^School of Exercise, Biomedical and Health Sciences, Edith Cowan University, Joondalup, WA, Australia

**Keywords:** metals, brain, sex hormones, AbetaPP transgenic mouse, leuprolide acetate, ovariectomy, copper

## Abstract

Alterations in brain metal ion homeostasis have been reported with aging and are implicated in the pathogenesis of neurodegenerative diseases. To assess whether age-related changes in hypothalamic-pituitary-gonadal (HPG) hormones might be involved in modulating brain metal ion homeostasis, we treated 7.5-month intact, sham-ovariecomized and ovariectomized C57B6SJL mice with vehicle or leuprolide acetate (for 9-months) to differentiate between whether sex steroids or gonadotropins might modulate brain metal ion concentrations. Unlike other aging mammals, there was no increase in plasma luteinizing hormone (LH) and follicle-stimulating hormone (FSH) concentrations following estropause in mice, suggesting there was sufficient residual production by the follicle depleted ovary, of sex steroids like estrogens and protein hormones like the inhibins, in order to suppress pituitary LH/FSH production. Castration on the other hand induced significant increases in circulating LH and FSH. Modulation of plasma sex steroid and gonadotropin levels did not significantly alter the concentrations of brain metals tested (Fe, Zn, Cu, Mn, Co, Ni, Al, Li), although there was a tendency for a decrease in all brain metals following ovariectomy (low estrogens and progesterone, high gonadotropins), a response that was reversed with leuprolide acetate treatment (low sex steroids, low gonadotropins). Brain Cu concentration was the only metal correlated with plasma LH (−0.37, *n* = 30, *p* < 0.05) and FSH (−0.42, *n* = 29, *p* < 0.01). This study demonstrates that sex hormones do not markedly alter brain metal ion homeostasis, unlike previously reported studies of circulating metal ion homeostasis. The role of gonadotropins in regulating metal ion homeostasis does however warrant further study.

## Introduction

Metal ion homeostasis is largely unaltered in mice of reproductive age [e.g., (1)]. However, studies in mice and humans show that the concentrations of Fe, Cu and Co increase in several tissues including the brain during normal aging (1–7), while the concentration of brain Mn decreases (1) and the concentration of Zn either remains unchanged or shows a slight decrease (1, 4, 6, 8, 9). These results imply an age-related change in the homeostatic mechanisms regulating brain metal ion concentrations.

Metal ions also have been postulated to play a role in the pathogenesis of age-related neurological disorders such as Alzheimer's disease [AD; (10, 11)]. Cu, Zn, and Fe are concentrated in and around amyloid plaques in the AD brain (12), and high levels of Zn (13) and Fe (14) have been reported in the amyloid plaques of the Tg2576 mouse model of AD. Numerous reports also have demonstrated transition metal imbalances in the parenchyma of the AD brain, such as decreased Cu, and increased Fe, Zn, Mn (15–20) and Al [e.g., (21)].

Aging is associated with marked changes in hormones of the hypothalamic-pituitary-gonadal (HPG) axis, which are implicated in the etiology of AD (22, 23). These changes include the loss of gonadal sex steroid and inhibin production, and marked elevations in hypothalamic gonadotropin-releasing hormone (GnRH) secretion (24) and serum (25–27) and CSF gonadotropin levels (28). The more abrupt changes in HPG axis hormones in women at menopause compared with men during andropause has been proposed as an explanation for the increased prevalence of AD in women compared with men [~2:1 ratio; (23)].

Evidence supporting hormonally-induced changes in metal ion homeostasis come from early studies demonstrating that Cu (but not Zn) is positively correlated with plasma estrogen and progesterone during the estrous cycle, after ovariectomy and during administration of these sex steroids to female Sprague-Dawley rats (29). An impact of circulating metals on brain metal homeostasis is suggested by 1) increases in Zn concentrations in the brain (minus cerebellum) and cerebellum, and increased Cu in the cerebellum, after both estrone and testosterone treatment of mice (30) and 2) significant decreases in immunohistochemically detectable Fe in the cerebral cortex during diestrus (low estradiol) compared to proestrus when estradiol levels are highest (31). In this latter study, there was a shift in Fe localization from neurons to vascular endothelial cells and oligodendrocytes in the transition from diestrus to proestrus. Additional evidence supporting a role for sex hormones in modulating brain metals is indicated by gender differences in Zn levels in the brains of aging mice (1) and lower Cu and Mn levels in the brains of female mice compared to male mice at any age (32). The total amount of Fe present in the serum, liver and the substantia nigra also fluctuates during the estrous cycle in female rats (33), as does hypothalamic Zn and Cu that rise between proestrus and estrus and fall again at diestrus (34). These workers also demonstrated that castration decreased the concentrations of Fe, Cu and As in both pituitary and hypothalamus, while zinc concentrations rose. Male rats also had lower pituitary concentrations of Fe, Cu, Zn, As, and Rb than cycling females.

Together these data raise the possibility that changes in the concentration of circulating HPG hormones can influence the intercellular transport of metals in the brain. We therefore undertook this study to assess which HPG hormones might be involved in modulating brain metal ion homeostasis. We modulated serum hormone levels with both ovariectomy and leuprolide acetate, both alone and in combination to differentiate between whether sex steroids or gonadotropins might modulate brain metal ion concentrations. Modulation of plasma sex steroid and gonadotropin levels did not alter any brain metals tested, although there was a tendency for a decrease in brain Cu following ovariectomy (low estrogens, high gonadotropins) which was reversed with leuprolide acetate treatment (low sex steroids, low gonadotropins) of these mice. Our results indicate the importance of metal ion homeostasis for normal brain function.

## Methods

### Animals

Female C57B6SJL mice (Taconic, Germantown, NY) were housed under a 12-h light, 12-h dark cycle, with food and water available *ad libitum*. All procedures performed on animals were reviewed and approved by the Animal Resource Center of Case Western Reserve University before the initiation of the study.

### Experimental Design

Due to the short half-life and expense of luteinizing hormone (LH) and follicle-stimulating hormone (FSH), administration of these gonadotropins via injection or pumps is not feasible for long-term studies. We therefore used ovariectomy to induce elevated levels of blood LH and FSH, a procedure that mimics the abrupt loss of estrogen following menopause. Conversely, leuprolide acetate (leuprolide) was administered intramuscularly to lower blood gonadotropin levels. Leuprolide is a synthetic analog of gonadotropin-releasing hormone (GnRH) acting mainly on the pituitary gland. Continuous treatment produces initial stimulation of the pituitary and an increase in blood gonadotropins, followed by a suppression of blood gonadotropins and sex steroids to castrate/post-menopausal levels within ~ 1 week due to suppression of GnRH receptor. In females, both ovarian estrogen and androgen synthesis are inhibited.

In this study, female C57B6SJL mice at ~7.5 months of age (prior to amyloid deposition in the Tg2576 mice) were allocated to 5 different groups: either left intact and treated with vehicle, intact and treated with leuprolide, sham-ovariectomized, ovariectomized or ovariectomized and treated with leuprolide for 9 months (until ~16.5 months of age). Table 1 summarizes the experimental design and predicted effects of each treatment on blood hormone levels. In the ovariectomized vehicle group, blood LH and FSH level will be high but estrogen levels low; in the ovariectomized leuprolide-treated group, blood LH, FSH and estrogen level will be low. This experimental design allows differentiation between the effects of gonadotropins and sex steroids on brain metals.

**Table 1:** Summary of treatment groups for hormonal modulation of sex steroids and gonadotropins.

**Group name**	**LH and FSH level c.f. control**	**Estrogen level c.f. control**	**Number of mice**	**Age at euthanasia (month)**
Control -Vehicle	Normal	Normal	6	16
Leuprolide	Low	Low	6	16
Sham OVX	Normal	Normal	6	16
Sham OVX + Vehicle	High	Low	6	16
OVX + Leuprolide	Normal	Low	6	16

### Treatments

Leuprolide acetate (Lupron Depot, TAP Pharmaceuticals Inc., Lake Forest) is a GnRH agonist that lowers LH/FSH levels by desensitizing GnRH receptor signaling that subsequently leads to decreased LH/FSH secretion and decreased blood sex steroid levels. Leuprolide is mixed with a diluent in a prefilled dual-chamber syringe containing sterile lyophilized microspheres, to form a suspension. Leuprolide is gradually released from the microspheres after injection over a 4-week period. Animals were injected with vehicle or leuprolide acetate (1.5 mg/kg) biweekly during the first month and monthly thereafter for 9 months. Mice were ovariectomized using a bilateral dorsal approach at 29 weeks of age. After the wounds healed 1 week later, mice were injected with either vehicle (saline) or leuprolide acetate (1.5 mg/kg). Animals were weighed monthly after saline or leuprolide injection.

### Tissue Collection

Blood was collected at the commencement of the experiment (~7 months of age) and at the end of the 1st and 3rd month from an orbital sinus after mice were anesthetized with an IP injection of mouse anesthesia cocktail (0.1 mL/25 g containing ketamine HC1 15 mg (100 mg/mL) 0.15 mL; xylazine HC1 3 mg (20 mg/mL) 0.15 mL; acepromazine 0.5 mg (10 mg/mL) 0.05 mL; and sterile saline 1.4 mL). The final bleed at 9 months was collected by heart puncture (see below). Blood was collected from animals prior to their next dose of leuprolide. Blood (100–400 μL) was collected into EDTA-coated tubes which were centrifuged at 1,000 *g* for 3 min. at 4°C, followed by centrifugation at 5,000 *g* for 10 min. prior to the collection of plasma which was stored -80°C.

Mice were sacrificed at 16 months of age with an injection of sodium barbital (392 mg/mL, 50 μL each, IP). Blood was collected at this time by heart puncture, and the animals were then perfused with phosphate buffered saline (0.01 M, pH 7.4, Gibco, Invitrogen, Carlsbad, CA) and decapitated. The brain was removed from the skull and one hemisphere frozen in liquid nitrogen and stored at −80°C for metal ion analyses.

### Hormone Assays

The levels of LH and FSH were assayed by radioimmunoassay at the National Hormone and Peptide Program (Torrance, CA).

### Metal Ion Analysis

A brain hemisphere without the cerebellum and olfactory bulb was weighed and then homogenized in Tris buffer, pH 7.6 (20 mM; ultra pure Tris; MP Biomedicals, Aurora, OH) containing EDTA (1 mM), EGTA (1 mM) and a protease inhibitor cocktail (all from Sigma, St. Louis, MO). Following protein analysis by using the BCA assay (Pierce, Rockford, IL), approximately one third of the homogenate (~150 μL) was prepared for metal analysis as previously reported (1). Briefly, samples were mixed with 150 μL of 70% metal grade nitric acid (Fisher Scientific, Pittsburgh, PA) and incubated at 80°C in a water bath until the solution was clear. After cooling to room temperature, ~150 μL of 30% H_2_O_2_, was added and after the effervescence ceased, the samples were incubated at 70°C for 15 min. and then cooled to room temperature. The final volume was brought to 5 mL with 1% metal grade nitric acid. Samples were then analyzed for Fe, Zn, Cu, Mn, Co, Ni, Al and Li using inductively coupled plasma-mass spectrometry (ICP-MS) at the Soil & Plant Analysis Laboratory, University of Wisconsin-Madison. The metal ion contents of the buffer solutions were subtracted from the samples. VG PlasmaQuad PQ2 Turbo Plus ICP-MS was the apparatus used. Samples in solution were converted to aerosols via a nebulizer, and the aerosols are transported to the inductively coupled plasma, a high temperature zone (8,000–10,000°C). The analytes were heated (excited) to the respective ions. In a quadrupole mass filter, ions are separated based on their mass-to charge and the ion intensities at different masses were measured (mass spectrometry). Ion intensities are proportional to the respective concentrations of analytes in the aqueous sample. The quantification was an external multi-point linear calibration by comparing the ion intensity of an unknown sample with that of a standard sample. Multielement calibration standard solutions were prepared from single- and multielement primary and/or in-house working standard solutions. Rhodium (Rh) was used as an internal reference standard. Containers (bottles, vials, etc) were soaked in 10% nitric acid overnight and rinsed with de-ionized water several times before use.

### Statistical Analyses

Two-way ANOVA with repeated measures of one factor was used to assess statistically significant differences between treatment groups and temporal changes for plasma hormones and animal weights. Tukey's *post-hoc* tests were carried out to compare specific groups using the GraphPad Prism Software Version 5.0 (San Diego, CA). *t*-test and one-way ANOVA were used to analyze statistically significant differences between treatment groups for brain metals. Experiments on the vehicle and leuprolide treated group were performed at a different time to the sham operated, ovariectomized, and ovariectomized and leuprolide treated groups due to the availability of animals. To avoid potential systemic errors between these two subgroups, we performed separate statistical analyses. A correlation matrix of the endpoints (plasma hormones, body weight and brain metals) measured in this study was performed using Statview *v*5, SAS Institute.

## Results

### Mouse Weights

**Figure 1:**
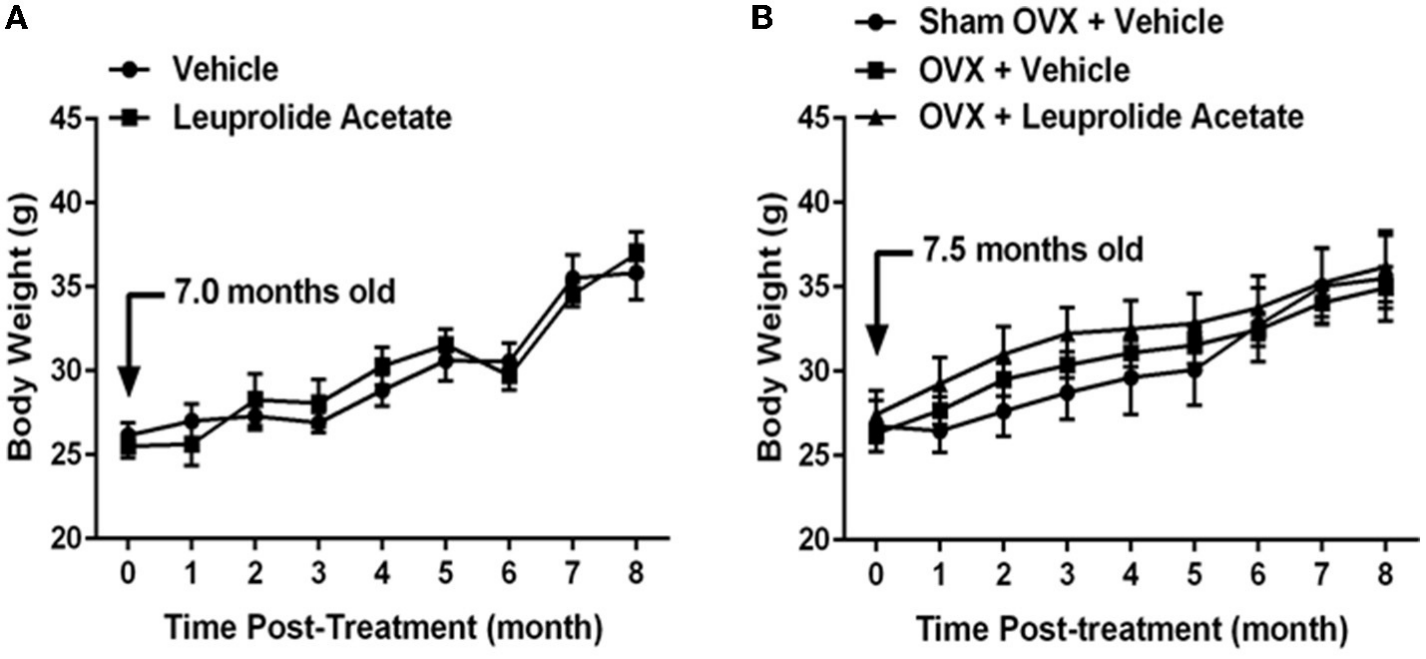
Changes in body weight with aging in ovariectomized and leuprolide acetate treated mice. **(A)** Female C57B6JL mice at the age of 7.5 months were treated either with vehicle (saline) or leuprolide acetate (1.5 mg/kg) monthly for 9 months and body weights determined each month. **(B)** C57B6JL mice at the age of 7.0 months were sham operated or ovariectomized. Ovariectomized animals were either treated with vehicle (saline) or leuprolide acetate (1.5 mg/kg) monthly for 9 months and body weights determined each month. Results are presented as mean ± SEM, *n* = 6.

The weights of control animals and leuprolide treated animals progressively increased 37% and 45 %, respectively, from 7.5 months of age (0 months post-treatment) to 15.5 months (8 months post-treatment) of age (Figure 1A). No significant difference in mouse weights was found between vehicle and leuprolide treated groups, but time was found to be a factor that influenced weights significantly. Control mice reached significantly heavier weights earlier (12.5 months) than leuprolide treated mice (14.5 months).

The weights of sham-operated, ovariectomized and ovariectomized plus leuprolide treated animals progressively increased 33, 33, and 32%, respectively, from 7 months (0 months post-treatment) to 15 months (8 months post-treatment) of age (Figure 1B). There was no significant difference in weights among sham operated, ovariectomized and ovariectomized and leuprolide treated groups, but time was found to be a factor that influenced weights significantly. Ovariectomized and ovariectomized plus leuprolide treated mice reached significantly heavier weights earlier (9 months) than sham-operated mice (11 months).

### Plasma Hormones

**Figure 2:**
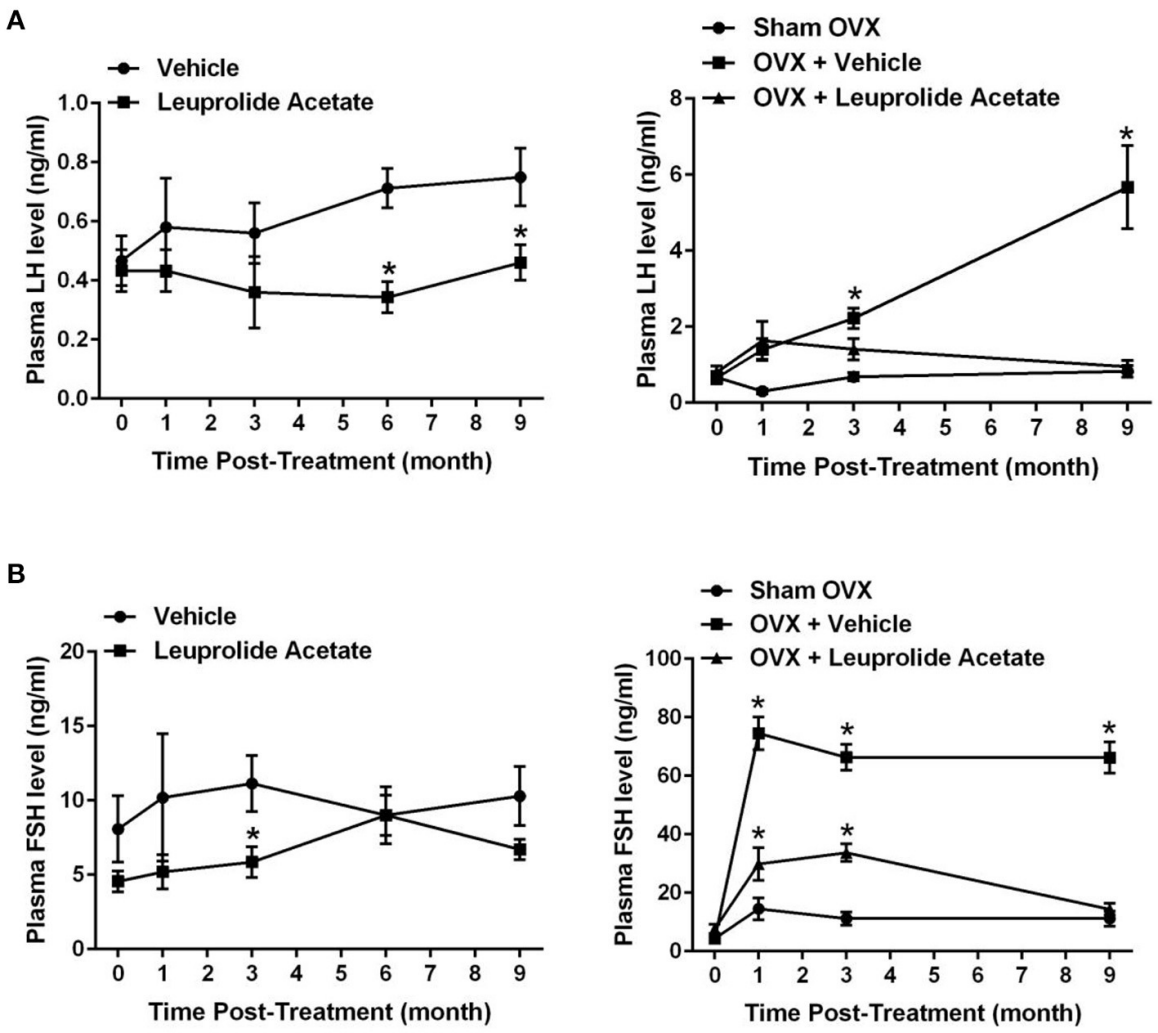
Changes in plasma LH and FSH with aging in ovariectomized and leuprolide acetate treated mice. Female C57B6JL mice at the age of 7.5 months were treated either with vehicle (saline) or leuprolide acetate (1.5 mg/kg) monthly, blood was taken at 0, 1, 3, 6, and 9 month and LH **(A)** and FSH **(B)** concentrations were assayed *via* RIA. C57B6JL mice at the age of 7.0 months were sham operated or ovariectomized. Ovariectomized animals were either treated with vehicle (saline) or leuprolide acetate (1.5 mg/kg) monthly for 9 months and blood samples collected at 0, 1, 3, and 9 month, and LH (B) and FSH (D) levels assayed *via* RIA. Results are presented as mean ± SEM, *n* = 6, *p < 0.05.

To confirm that ovariectomy and leuprolide acetate treatments modulated blood hormone levels, plasma collected from mice at 0, 1, 3, and 9 months (and also 6 months for vehicle and leuprolide treated groups) post-treatment were analyzed for LH and FSH concentrations (Figures 2A,B; respectively). There was a gradual 38% increase in the concentration of plasma LH from 0.47 ± 0.085 at 7.5 months of age (time 0 months) to 0.75 ± 0.09 (mean ± SEM; *P* < 0.05; *n* = 6) at 16.5 months of age (time 9 months). This increase in plasma LH, albeit non-significant, is consistent with the loss of negative feedback by 17 β-estradiol on the hypothalamus with the decline in reproductive function in female mice at this time (35, 36). The concentration of plasma LH in the leuprolide acetate treated mice was the same as vehicle treated mice at time 0, and remained at this lower concentration with the difference from the vehicle treated group becoming significant at the 6 month time point (Figure 2A).

The concentration of plasma LH did not change between 7 and 16 months of age in sham ovariectomized animals (Figure 2A). Ovariectomy induced a significant and progressive increase in the concentration of plasma LH such that by 9 months post-surgery (16 months of age), plasma LH concentrations were 8.6-fold elevated compared to time 0 (7 months of age; 5.7 ± 1.1 vs. 0.66 ± 0.16, respectively; *P* < 0.05; *n* = 6). Leuprolide acetate treatment of ovariectomized mice suppressed the surge in the concentration of plasma LH, and suppression was not obvious at 1 month post-treatment, but became significant at 3 months post-treatment and more so at 9 months post-treatment where the concentration of plasma LH was reduced to the same level as sham OVX mice.

Plasma FSH levels in vehicle treated mice fluctuated, but showed an overall trend toward an increase (Figure 2B). The average level of FSH increased about 60% at 16.5 months of age, but the increase was not statistically significant. Like plasma LH, this increase in plasma FSH is consistent with a loss of negative feedback by 17β-estradiol on the hypothalamus with the decline in reproductive function in female mice at this time (37). Leuprolide acetate treatment significantly suppressed the concentration of FSH in plasma at 3 (5.86 ± 1.03 vs. 11.14 ± 1.88; *P* = 0.05; *n* = 8–9) and 9 (6.7 ± 0.69 vs. 10.3 ± 1.98; *P* = 0.05; *n* = 8) months post-treatment, but not at 1 (5.20 ± 1.16 vs. 3.33 ± 1.33; *P* = 0.05; *n* = 5) or 6 (9.00 ± 1.34 vs. 9.00 ± 1.92; *P* = 0.05; *n* = 5–6) months post-treatment (Figure 2B). In sham ovariectomy mice, there was an initial significant 3-fold increase in plasma FSH at 1 month (14.5 ± 3.71 *P* < 0.05; *n* = 6) and the concentration of FSH remained at this level to 9 months post-surgery (11.24 ± 2.58; *n* = 6). Ovariectomy induced a significant 17.1-fold increase in the concentration of plasma FSH from 4.36 ± 0.66 pre-surgery to 74.5 ± 5.60 (*P* < 0.05; *n* = 6) at 1 month post-surgery and the concentration of plasma FSH remained at this level to 9 months post-surgery. Leuprolide acetate treatment of mice that were ovariectomized partially suppressed the surge in plasma FSH at 1 month (29.83 ± 5.67; *n* = 6) and 3 month (33.69 ± 3.01; *n* = 6) and completely suppressed plasma FSH concentrations to sham ovariectomy levels by 9 months post-surgery (14.37 ± 2.02; *n* = 6).

Overall, these results indicate that leuprolide acetate suppresses plasma LH and FSH in aging mice, and that the ovariectomy induced surge in plasma LH and FSH is suppressed by leuprolide acetate.

### Brain Metals

**Figure 3:**
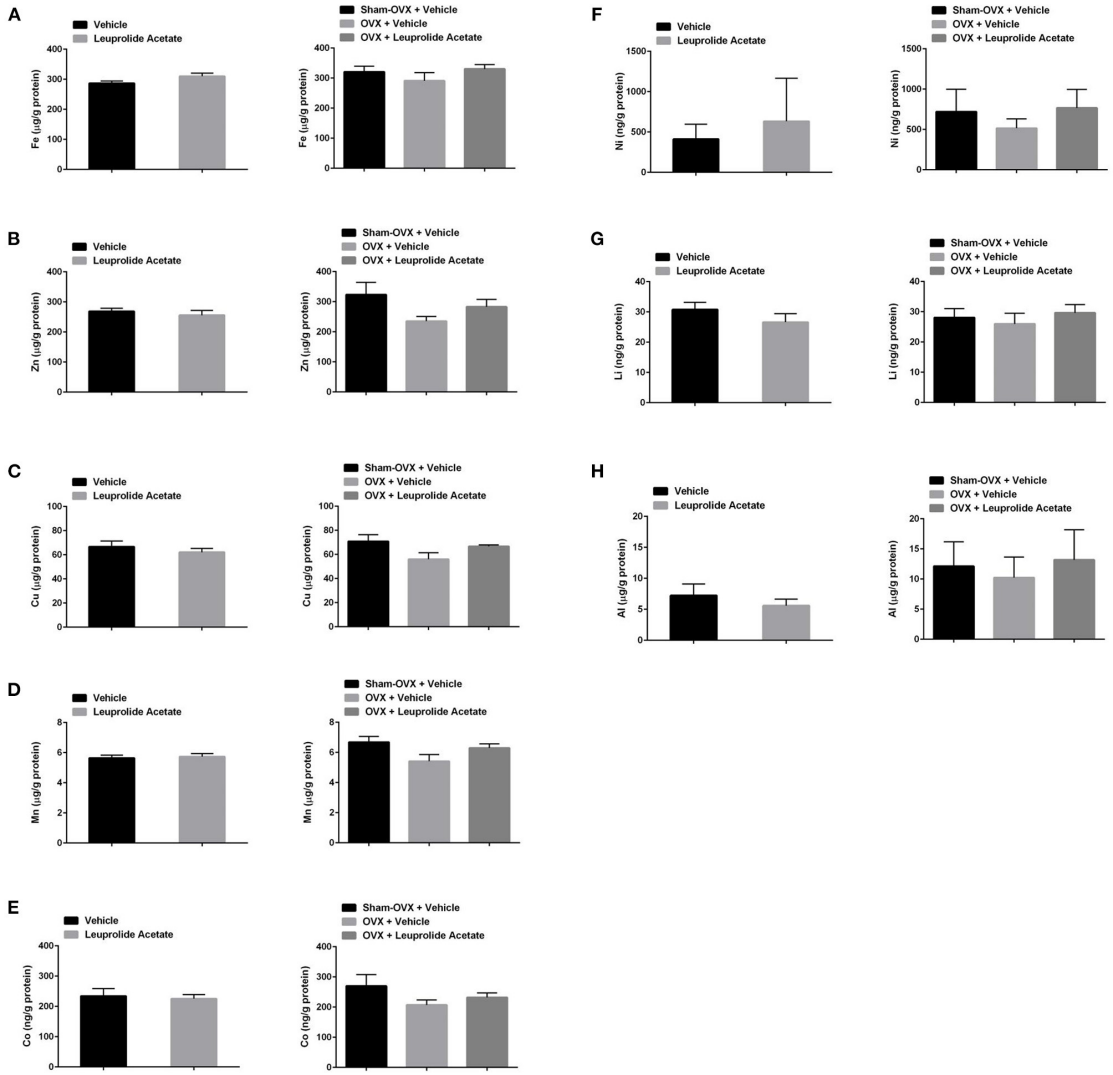
Changes in brain metal ion concentrations with ovariectomy and leuprolide acetate treatment of mice. Mice as described in Figures 1, 2 were sacrificed after 9 months of treatment, and their brains collected for metal analysis with ICP-MS. Samples were corrected for trace amounts of metals from buffer, and the concentration of metal ion was normalized to total protein in the samples. **(A)** Fe, **(B)** Zn, **(C)** Cu, **(D)** Mn, **(E)** Co, **(F)** Ni, **(G)** Li, and **(H)** Al. Results are presented as μg/mg or ng/mg protein; mean ± SEM, *n* = 6.

The concentration of metals in the brains of 16.5 month old mice in control, leuprolide, and 16 months old sham, ovariectomized and ovariectomized + leuprolide groups is shown in Figures 3A–H. Leuprolide acetate did not induce a significant change in the concentrations of any metals tested. There was however a trend toward a decrease in the concentration of Cu (about 7%), Zn (about 5%), but these changes were not statistically significant. Ovariectomy also did not significantly alter brain metal ion concentrations although there was a trend to a decrease with all metals, with the decrease in brain Cu being most significant (P = 0.087). These decreases were partly reversed by leuprolide acetate treatment for all metals, but again were not statistically significant (Figure 3C). Since leuprolide acetate decreases both plasma LH and FSH after 9 months of treatment (Figures 2A,B), the decrease in brain metals may be due to either decreased LH receptor or FSH receptor signaling, or increased GnRH receptor signaling. Since FSH receptors have not been identified in the brain (data not shown), it is unlikely that FSH is mediating changes in the concentrations of these metal ion concentrations.

To further examine changes in plasma LH and FSH with brain metal ion concentration, we performed a regression analysis (Table 2). The concentrations of plasma LH and FSH were highly positively correlated (*r* = 0.848, *n* = 29, *P* < 0.001), indicating that LH and FSH concentrations move in concert with one another. The concentration of brain Cu was significantly negatively correlated with LH (-0.370, *n* = 30, *P* < 0.05) and FSH (-0.422, *n* = 29, *P* < 0.05). No other brain metals were correlated with LH or FSH. However, the concentrations of Fe, Zn, Cu, Mn and Co in the brain were all significantly positively correlated with one another (P ≤ 0.01, *n* = 29/30). Other significant positive correlations included Li with Fe, Cu, Mn and Co; Ni with Zn, Co and Al, and Co with Mn. There was no correlation between body weight and either plasma hormones or brain metals (data not shown).

**Table 2:** Correlation analysis of plasma hormone and brain metal ion concentrations.

	**LH**	**FSH**	**Fe**	**Zn**	**Cu**	**Mn**	**Co**	**Ni**	**Al**	**Li**
LH	1									
FSH	0.85^***^	1								
Fe	−0.19	0.16	1							
Zn	−0.23	−0.26	0.64^***^	1						
Cu	−0.37^*^	−0.42^*^	0.62^***^	0.51^**^	1					
Mn	−0.27	−0.28	0.88^***^	0.74^***^	0.68^***^	1				
Co	−0.25	−0.20	0.62^***^	0.57^**^	0.74^***^	0.68^***^	1			
Ni	−0.01	0.11	0.34	0.43^*^	0.17	0.24	0.58^***^	1		
Al	−0.01	0.12	−0.17	0.25	−0.28	−0.09	0.06	0.66^***^	1	
Li	−0.01	−0.13	0.55^**^	0.35	0.46^**^	0.47^**^	0.46^*^	0.17	−0.28	1

* *P <0.05*;

** *P <0.01*;

*** *P <0.001*.

## Discussion

### Circulating Sex Hormone Concentrations Associated With Aging, Ovariectomy and Lupron Treatment in Female Mice

We observed a non-significant increase in plasma LH and FSH concentrations between 7.5 and 16.5 months of age in ovary intact and Sham OVX female C57B6SJL mice. This is contrary to other mammals, where significant elevations in both LH and FSH are observed following estropause/menopause (24, 38–41). These results suggest that there was sufficient residual production by the follicle depleted ovary, of sex steroids like estrogens and protein hormones like the inhibins, in order to suppress elevations in circulating LH/FSH that are observed in other species (42, 43). In mice, ovariectomy should therefore not be equated with estropause with respect to the interpretation of biochemical and physiological endpoints resulting from these different forms of HPG axis dysregulation. As in other species, leuprolide acetate lowered blood gonadotropin concentrations in the aging intact and ovariectomized female mouse [Figure 2; (44–46)].

Ovariectomy induced a gradual increase in plasma LH over 9 months between 7 and 16 months of age, while plasma FSH levels increased dramatically within 1 month of ovariectomy and remained elevated through to 16 months of age. The abrupt increase in plasma FSH but gradual increase in plasma LH following ovariectomy is consistence with previous findings in ovariectomized mice (35, 36) and many other species including sheep where LH and FSH are thought to be subjected to differential regulation after ovariectomy (47). Alternatively, it is possible that since blood was collected prior to the next dose of leuprolide, that the previously injected leuprolide was becoming ineffective at suppressing plasma LH/FSH. However, this is difficult to reconcile with the fact that leuprolide treatment decreased plasma LH and FSH in the ovariectomized animals (Figure 2). Irrespective, these results indicate that the pituitary is responding to the loss of sex steroid feedback, particularly following ovariectomy, with the pituitary capable of producing large quantities of gonadotropins. Overall, these results indicate that ovariectomy is not equivalent to estropause with respect to hormonal changes.

### Hormonal Regulation of Body Weight

Although there was no difference between the weights of sham-operated, ovariectomized and ovariectomized plus leuprolide treated animals at 15 months (8 months post-treatment; Figure 1B), ovariectomized and ovariectomized plus leuprolide treated mice reached significantly heavier weights earlier (9 months) than sham-operated mice (11 months), suggesting that the loss of estrogens rather than the elevation in gonadotropins was responsible for this faster weight gain. This is supported by the more rapid gain in body weight by the sham OVX group at 12.5 months of age that parallels the loss of reproductive function (loss of circulating estradiol) at this time in female mice. A similar increase in weight gain was observed for vehicle treated and leuprolide acetate treated mice between 13.5 and 14.5 months of age suggesting that hormones other than sex steroids and gonadotropins may regulate the weight gain observed with ovariectomy/estropause (Figure 1A).

### Hormonal-Induced Modulation of Brain Metals

Our study demonstrates a possible negative relationship between circulating gonadotropin concentrations and brain Cu concentration (Figure 3 and Table 1). Ovariectomy, which induces an increase in GnRH, LH and FSH secretion, but a decrease in sex steroid secretion, correlated with a trend toward an increase in brain Cu concentration, suggesting that these hormonal changes either decrease neuronal Cu uptake or increase neuronal Cu efflux. Interestingly, leuprolide acetate treatment of ovariectomized mice prevented the ovariectomy-induced decline in brain Cu levels, suggesting that either LH, FSH or GnRH receptor signaling may play an important role in Cu homeostasis in the brain. Estrogen along with progestogen has been shown to increase serum Cu, ceruloplasmin and brain Cu levels in humans and rats, while the hepatic levels of Cu decrease (48).

It is not clear if these hormonal changes redistribute metals within the brain, or alter synaptic metal ion release. Both synaptic Zn and Cu are released during normal synaptic transmission. Zn, like glutamate, is released during paroxysmal activity (49–51). Zn is released in a calcium-dependent manner from the hippocampal mossy fiber terminals during spontaneous activity (52), after stimulation evoked electrically (53) after administration of K^+^ and after administration of kainic acid (51, 54). The physiological purpose of such high Zn concentrations in the hippocampus is unclear but some workers have proposed that this large trans-synaptic movement of Zn may have a normal signaling function (49, 55) and be involved in long-term potentiation i.e., in the processing of memory formation (56). Changes in behavior of multiple channels and receptors (57, 58), in particular inhibition of NMDA receptors (59) have been reported.

It has been shown that extracellular Cu modulates the secretory function of peptidergic neurons and plays an important modulatory role in the central nervous system. Cu is released in a Ca-dependent manner from vesicles of peptidergic neurons following K^+^-induced depolarization of hypothalamic tissues *in vitro* (60, 61). The release of Cu (bound to an intracellular chelator) during depolarization is thought to oxidize thiols of the gonadotropin-releasing hormone (GnRH) granule in an oxygen dependent manner thereby altering the permeability of granule membranes and mediating the release of GnRH and α-melanotropin from hypothalamic granules of explants of the median eminence area (MEA) (62–64). These alterations in membrane permeability are specific for complexed Cu, and to a lesser extend Zn (62, 63). Released Cu might also act to complex GnRH, since injection of rats with Cu complexed GnRH brings about a high release of LH and even higher release of FSH compared with uncomplexed GnRH or metal ion alone in ovariectomized, estradiol, and progesterone pretreated rats (65). The Ni complex showed a similar although lesser effect. Although the Zn complex had similar potency to free GnRH, it had higher FSH-releasing ability (65). How the metal ion and release of gonadotropins interplay with each other remains to be determined.

Brain Fe concentrations trended lower following ovariectomy while leuprolide partially restored decline brain Fe concentrations, although these changes following our chronic experimental manipulations were not significantly different (Figure 3 and Table 1). Our results suggest that like Cu, either LH or GnRH receptor signaling may play an important role in Fe homeostasis in the brain. One previous study using an immunohistochemical method of detection noted a modest but significant increase in histochemically detectable Fe in the cerebral cortex during proestrus, when serum estradiol and gonadotropin levels are transiently upregulated (31). Serum progesterone levels which peak during proestrus also might explain the increase in histochemically detectable Fe in the cerebral cortex at this time. There is evidence that the expression of proteins involved in Fe metabolism fluctuate during the oestrous cycle, 1) endothelial cells in chick brain express hen oviduct transferrin receptor (66), which can bind transferrin, ferritin and lactoferrin (67, 68). Expression of this receptor is induced by estrogen (69), so its expression should be maximal during proestrus, 2) lactoferrin protein and mRNA expression in mouse genital tract are positively correlated with estradiol levels (70) and lactoferrin gene expression is induced by estrogen in the reproductive tracts of rats, hamsters and primates (71, 72). Progesterone may antagonize the effects of estrogen, particularly with respect to lactoferrin expression in the mouse reproductive tract (73).

Like Cu and Fe, ovariectomy tended to induce a decrease while leuprolide partially restored brain Zn levels, although again these changes were not statistically significantly different (Figure 3 and Table 1). Our results suggest that like Cu and Fe, either LH, or GnRH receptor signaling may play an important role in Zn homeostasis in the brain. This result is in contrast to those of Lee and colleagues, where ovariectomy of 5-month old female mice slightly increased total and hippocampal synaptic vesicle Zn levels, and estrogen replacement returned these Zn concentrations to control levels (74). These changes appeared to be mediated by estrogen-induced decreases in Zn transporter 3 (Znt3) expression and the delta subunit of ad aptor protein complex (AP)-3, which modulates the level of Znt3 levels. Estrogen has been shown to elevate liver Zn levels (75). Since both leuprolide treatment and estradiol treatment have the effect of rebalancing the ratio of estrogens to gonadotropins, the ratios of these hormones may be a better predictor of Zn influx vs. Zn efflux in the brain compared to either hormone concentration alone.

In the context of the preceding observations, our present data support the possibility that fluxes in serum reproductive hormones during the estrous cycle could influence the intercellular transport of certain metals in the brain. In our experiments, ovariectomy was performed 9 months prior to and leuprolide administered for 9 months prior to collection of brain tissue for metal ion analysis. Homeostatic mechanisms may have compensated for changes in brain metals during these chronic experimental manipulations. It will be interesting to determine brain metal ion concentrations in a short-term study.

### Metal Ion Agonism/Antagonism

A number of studies have demonstrated that certain metals move in the same direction or in the opposite direction to one another. For example, Zn and Cu are biologically antagonistic; alterations in Zn metabolism directly impact Cu metabolism and vice versa (76). *In vitro* studies also indicate the antagonistic relationship of Zn and Cu; Aß binds equimolar amounts of Cu^2+^ and Zn^2+^ at pH 7.4, but Cu^2+^ displaces Zn^2+^ from Zn^2+^:Aß complexes under acidic conditions [pH 6.6; (77)]. In contrast to previous literature, we observed a positive correlation between brain Cu and Zn (Table 2; *r* = 0.51).

Zn and Fe antagonism (78) also has been demonstrated from nutritional studies; Fe reduces the absorption of Zn across the gut (79–82). The interdependence of Fe and Zn in the brain also has been studied. In contrast to the gut, Fe and Zn appear to have an agonistic relationship. Fe deficient rats administered Fe + Zn have significantly higher numbers of Mossy fiber (NF) cells than either metal ion treatment alone, suggesting that since MF of the hippocampus are thought to be involved in LTP, this agonistic relationship is important for neurotransmission (83). We also noted a positive relationship between Fe and Zn (Table 2; *r* = 0.64).

### Neurological Implications of Hormonal Induced Changes in Metal Ion Homeostasis

Given the normal physiological functions of brain metals, dysregulated metal ion homeostasis induced by hormonal, nutritional or genetic factors could be very detrimental to neuron health. Although gross changes in metals in a brain hemisphere were relatively small in our study following dysregulation of the HPG axis *via* ovariectomy and/or GnRH superagonist treatment (Figure 3), previous studies noted above demonstrate significant changes in metals between sex, following castration and during the estrous cycle suggesting that hormone-induced metal ion changes are important for normal neurotransmission and other functions. One possible explanation for the small global changes in metals in our study is that adaptive changes in brain sex hormones may mask metal ion changes induced by endocrine changes in sex hormones with aging or following ovariectomy. Previous studies indicate regional differences in metals throughout the brain (see Introduction) and association with AD neuropathology (84, 85). Interestingly, regional changes in neurosteroids also have been reported in response to stress (86), while hydrocortisone has been reported to upregulate the expression of the potent metal ion binder metallothionein in the choroid plexus of rats (87). Thus, hormonal dysregulation of regional metal ion homeostasis could have implications for inducing neuroinflammation, AD neuropathology and neuroodegeneration (40, 88). Future studies directed at determining whether endocrine hormones elicit regional changes in metals and/or neurohormone concentrations resulting in neurodegeneration, are warranted.

## Conclusion

Our results demonstrate that changes in serum sex hormones, specifically gonadotropins, may alter the concentration of brain Cu (and perhaps Fe and Zn). However, all changes in the concentration of metals were small, and are indicative of the tight homeostatic mechanisms that exist in the brain for regulating brain metal ion concentrations required for numerous crucial cellular functions. Numerous correlations between brain metals also were identified, suggesting that primary metal changes induced by sex hormones might induce numerous other compensatory changes in brain metal concentrations.

## Data Availability Statement

The raw data supporting the conclusions of this article will be made available by the authors, without undue reservation.

## Ethics Statement

The animal study was reviewed and approved by Animal Resource Center of Case Western Reserve University.

## Author Contributions

TL performed the experiments, analyzed the data, and wrote the manuscript. AW helped with animal experimentation. RB helped with interpretation of results. CA conceptualized the study, assisted with metal assays and data analyses, and wrote the manuscript. All authors contributed to the article and approved the submitted version.

## Funding

This work was supported by funds from the Alzheimer's Association. This material is the result of work supported with resources at the William S. Middleton Memorial Veterans Hospital, Madison, Wisconsin.

## Author Disclaimer

The opinions expressed herein are those of the authors. The contents do not represent the views of the Department of Veterans Affairs or the U.S. Government. This is Geriatrics Research, Education and Clinical Center VA paper # 003-2022.

## Conflict of Interest

RB was employed by OTB Research. The remaining authors declare that the research was conducted in the absence of any commercial or financial relationships that could be construed as a potential conflict of interest.

## Publisher's Note

All claims expressed in this article are solely those of the authors and do not necessarily represent those of their affiliated organizations, or those of the publisher, the editors and the reviewers. Any product that may be evaluated in this article, or claim that may be made by its manufacturer, is not guaranteed or endorsed by the publisher.
